# Self‐Medication With Herbal Remedies During Pregnancy: Practices, Reasons, and Adverse Effects

**DOI:** 10.1155/sci5/1784792

**Published:** 2026-07-13

**Authors:** Mandana Sayadi Mank-Halati, Maryam Janatolmakan, Shahab Rezaeian, Sahar Omidi, Alireza Khatony

**Affiliations:** ^1^ Student Research Committee, Kermanshah University of Medical Sciences, Kermanshah, Iran, kums.ac.ir; ^2^ Infectious Diseases Research Centre, Health Policy and Promotion Institute, Kermanshah University of Medical Sciences, Kermanshah, Iran, kums.ac.ir

**Keywords:** herbal, pregnancy, prevalence, self-medication

## Abstract

**Background:**

Despite limited knowledge about the benefits and adverse effects of herbal remedies during pregnancy, self‐medication with these substances among pregnant women is increasing. The lack of precise data on the prevalence and reasons for this phenomenon poses a significant challenge to designing and implementing effective interventions to reduce it. Therefore, this study aimed to determine the prevalence of self‐medication with herbal remedies among pregnant women.

**Methods:**

In this cross‐sectional study, 166 pregnant women visiting health centers affiliated with primary healthcare facilities in Kermanshah, western Iran, were recruited using convenience sampling. Data were collected using a validated researcher‐designed questionnaire and analyzed using SPSS Version 18. Statistical analysis included descriptive statistics (frequency, percentage, mean, and standard deviation).

**Results:**

All 166 pregnant women with a mean age of 32.6 ± 5.1 years in this study reported using herbal remedies during pregnancy. The most common herbs consumed were fenugreek (89.8%), mint (71.1%), and cardamom (60.2%). The primary reasons for consumption were nausea and vomiting (12.7%), preventing neonatal jaundice (9%), and respiratory problems (6.6%). Despite this, 30.7% of women reported experiencing side effects such as abdominal pain (10.8%), headache (10.2%), and insomnia (3.6%) after consuming herbal remedies. Participants rated the effectiveness of herbal remedies as moderate (57.8%) and high (27.1%). However, only 30.7% were moderately satisfied and 28.9% were slightly dissatisfied with self‐medication with herbal remedies.

**Conclusion:**

The results of this study indicate a high prevalence of self‐medication with herbal remedies during pregnancy. Given the serious risks associated with the unsupervised use of these substances, including miscarriage and macrosomia, interventional measures to raise awareness among pregnant women about the dangers of self‐medicating with herbal remedies for both mother and fetus are essential. Utilizing mass media to disseminate this information is particularly crucial. Furthermore, conducting clinical trials to investigate the safety and efficacy of herbal remedies during pregnancy is recommended.

## 1. Introduction

Pregnancy is a physiological process associated with substantial anatomical, hormonal, and metabolic changes affecting nearly all body systems [1]. These changes are frequently accompanied by unpleasant symptoms such as nausea, vomiting, constipation, fatigue, and heartburn, which may prompt pregnant women to seek symptom relief through complementary therapies, including herbal remedies and over‐the‐counter medications [2]. In many cultures, herbal remedies are also used during pregnancy for the prevention or management of conditions such as miscarriage, anemia, osteoporosis, and common pregnancy‐related discomforts [3].

Self‐medication refers to the use of medicinal products without professional prescription or supervision, including nonprescription medications, reuse of previous prescriptions, and the consumption of herbal and traditional remedies [4]. This practice may occur based on personal beliefs, previous experiences, advice from family members or friends, or recommendations from traditional healers [5]. Self‐medication during pregnancy has become a growing public health concern worldwide because inappropriate use of medications and herbal products may expose both mother and fetus to preventable health risks [6, 7].

The prevalence of herbal remedy use during pregnancy varies considerably across countries and populations, ranging from 7% to 55% [8]. Recent evidence from African countries, particularly Ethiopia, has consistently demonstrated a high prevalence of self‐medication with herbal and conventional medicines among pregnant women [3, 9–11]. However, evidence from Middle Eastern countries, including Iran, remains limited. Differences in cultural beliefs, accessibility of herbal products, socioeconomic conditions, and traditional health practices may influence patterns of herbal remedy use across settings. Therefore, findings from other countries cannot be directly generalized to the Iranian context, indicating an important contextual research gap.

Pregnant women often perceive herbal remedies as “natural” and therefore safe during pregnancy [12]. Nevertheless, scientific evidence regarding the safety, efficacy, dosage, and potential adverse effects of many herbal products during pregnancy remains insufficient [12, 13]. Ethical concerns frequently limit the inclusion of pregnant women in clinical trials, resulting in inadequate evidence regarding the maternal and fetal safety of many pharmacological and herbal substances [14]. This knowledge gap may increase the likelihood of unsafe self‐medication practices during pregnancy.

The use of herbal remedies without adequate awareness of their potential risks may lead to serious maternal and fetal complications, including miscarriage, preterm labor, bleeding, uterine contractions, fetal developmental abnormalities, and drug–herb interactions [1, 2]. Physiological changes during pregnancy may alter the pharmacokinetics of medicinal substances, and compounds capable of crossing the placental barrier may adversely affect fetal development [15]. Despite these concerns, the use of herbal remedies among pregnant women continues to increase globally [12, 16], while regulatory oversight and evidence‐based counseling regarding herbal medicine use during pregnancy remain insufficient in many settings [10].

Beyond biomedical explanations, recent literature suggests that herbal remedy use during pregnancy may also reflect women’s behavioral responses to pregnancy‐related discomfort, uncertainty, and emotional stress. In this regard, such practices can be theoretically interpreted as informal coping strategies used by pregnant women to manage physical and psychological challenges, particularly in contexts where professional guidance or healthcare accessibility may be limited [17]. It is important to note that this coping perspective is used as a theoretical lens and was not empirically measured in the present study.

Although previous international studies have investigated self‐medication during pregnancy, most have focused on prevalence and associated factors, with limited attention to contextual and behavioral interpretations of herbal remedy use. Furthermore, most available evidence originates from African countries, particularly Ethiopia [3, 9–11], while evidence from Iran remains scarce. In particular, there is a lack of region‐specific data from western Iran, such as Kermanshah, where cultural reliance on herbal medicine is relatively strong. This highlights a clear research gap and the need for context‐specific evidence.

Given that pregnancy is a highly sensitive period in a woman’s life, self‐medication with herbal remedies may contribute to adverse maternal outcomes and fetal abnormalities. The birth of a child with congenital anomalies or long‐term disabilities can impose considerable emotional, social, and economic burdens on families and healthcare systems [11]. Therefore, identifying the prevalence, motivations, patterns, perceived effectiveness, and adverse effects of herbal remedy use during pregnancy is essential for designing effective preventive and educational interventions.

Accordingly, this study aimed to investigate self‐medication with herbal remedies among pregnant women attending healthcare clinics in Kermanshah, Iran. Specifically, the study examined the prevalence of herbal remedy use, commonly used herbal products, reasons for use, perceived effectiveness, satisfaction, and self‐reported side effects associated with herbal remedies during pregnancy. By addressing a context‐specific population in Iran and incorporating a broader behavioral interpretation of herbal use, this study contributes novel evidence to the existing literature on maternal self‐medication practices.

This study sought to answer the following questions:

• What is the prevalence of self‐medication with herbal remedies among pregnant women attending clinics in Kermanshah, Iran?
• What types of herbal remedies are commonly used during pregnancy?
• What are the reasons for herbal remedy use during pregnancy?
• What are the perceived benefits and motivations for herbal remedy use?
• What is the perceived effectiveness of herbal remedies used during pregnancy?
• What are the frequency and types of self‐reported side effects?
• What is the level of satisfaction regarding herbal remedy use during pregnancy?

## 2. Materials and Methods

### 2.1. Study Design

This descriptive cross‐sectional study was conducted from December 11, 2023, to April 19, 2024, at selected health centers affiliated with Kermanshah health centers in Kermanshah, Iran. The study adhered to the Strengthening the Reporting of Observational Studies in Epidemiology (STROBE) guidelines for reporting observational studies [18].

### 2.2. Participants and Sampling

The study population comprised all pregnant women attending healthcare centers affiliated with the urban health network of Kermanshah, Iran. The sample size was calculated to be 166, based on a similar study [16] and using the formula ${\left(z_{12-\alpha /}\right)}^{2}p\left(1-p\right)/d^{2}$, with a 95% confidence level and a margin of error equal to 0.1p0. Initially, Kermanshah City was divided into seven clusters based on municipal districts, and then one healthcare center was randomly selected from each cluster. Eligible participants were enrolled using a convenience sampling method. Inclusion criteria were as follows: Iranian nationality, literacy in reading and writing, and no history of physical illnesses (such as diabetes and high blood pressure) or mental illnesses (such as anxiety), based on self‐reporting. Incomplete questionnaires were the exclusion criterion.

### 2.3. Data Collection Instrument

Data were collected using a researcher‐developed questionnaire divided into two sections. The first section consisted of 11 items on sociodemographic and pregnancy‐related characteristics, including age, education level, occupation, residential area, number of pregnancies, history of miscarriage, current pregnancy status, gestational age, history of herbal remedies use during pregnancy, current use of herbal remedies during pregnancy, source of information about herbal remedies, and income level. The second section included 30 items on the types and frequency of herbal remedies used, reasons for self‐medication with herbal remedies, perceived effectiveness of herbal remedies, frequency and types of side effects experienced, and satisfaction with the use of herbal remedies.

The content validity of the questionnaire was assessed qualitatively. The questionnaire was reviewed by 12 faculty members specializing in nursing, midwifery, gynecology, and internal medicine. They were asked to evaluate the relevance, comprehensibility, and simplicity of each item and provide suggestions for improvement. The questionnaire was then revised based on their feedback. The reliability of the questionnaire was assessed using test–retest reliability, which showed good agreement (*r* = 0.74).

### 2.4. Data Collection

After obtaining ethical approval from the university ethics committee, the researcher visited the selected healthcare centers affiliated with the Kermanshah urban health network. Upon presenting an introduction letter to the authorities at each center, the researcher recruited pregnant women attending the centers. The study objectives were explained to potential participants, and those who agreed to participate were enrolled. Questionnaires were then distributed to the participants and collected after completion.

To ensure data quality, the following measures were taken: the questionnaire completion process was explained clearly and transparently to the participants, and their questions were answered; the confidentiality of responses and participant identities was emphasized; participants were given sufficient time to complete the questionnaires; appropriate statistical methods were used to manage missing data; and the research team reviewed the data entered into the software for accuracy and correctness at appropriate intervals and took corrective actions as necessary.

### 2.5. Declaration of AI Use

In preparing this manuscript, the authors made limited use of ChatGPT, an AI‐based language tool developed by OpenAI, exclusively for improving the clarity and flow of the language. All content generated with AI assistance was carefully reviewed, edited, and confirmed by the authors. The authors assume full responsibility for the accuracy, validity, and integrity of the manuscript. The AI tool was not involved in any aspect of the study design, data collection, data analysis, or interpretation of the results.

### 2.6. Statistical Analysis

Data were entered into SPSS Version 16.0 (SPSS Inc., Chicago, IL, USA). Descriptive statistics, including simple and relative frequency distributions, means, and standard deviations, were used to describe the data.

### 2.7. Ethical Considerations

The Ethics Committee of Kermanshah University of Medical Sciences approved the study under the Code IR.KUMS.REC.1402.316. The study objectives were initially explained to the participants, and their written informed consent was obtained. The anonymity of the questionnaires and confidentiality of the responses and participant identities were emphasized.

## 3. Results

### 3.1. Participants’ Characteristics

The response rate was 100%. The mean age of the participants was 32.6 ± 5.1 years (range: 21–45 years). The majority of participants were younger than 30 years old (*n* = 104, 62.7%), had a high school diploma or less (*n* = 94, 56.6%), were housewives (*n* = 147, 88.6%), and lived in urban areas (*n* = 142, 85.5%). Additionally, most participants had experienced 2 or fewer pregnancies (*n* = 142, 85.5%). Approximately 20.0% of the participants (*n* = 34) had a history of miscarriage. The majority of pregnancies were planned (*n* = 136, 81.9%). In 66.0% of the participants (*n* = 110), the gestational age was 25 weeks or less (Table 1).

**TABLE 1:** Characteristics of pregnant women who consume herbal remedies (*N* = 166).

Variables	*n* (%)
Age (years)	
≤ 30	104 (62.7)
> 30	62 (37.3)
Education	
Below high school	94 (56.6)
High school and above high school	72 (43.4)
Occupation	
Homemaker	147 (88.6)
Employed	19 (11.4)
Residence	
Urban	142 (85.5)
Rural	24 (14.5)
Number of gravidities	
≤ 2	142 (85.5)
> 2	24 (14.5)
History of abortion	
No	132 (79.5)
Yes	34 (20.5)
Pregnancy status	
Planned	136 (81.9)
Unplanned	30 (18.1)
Gestation age (weeks)	
≤ 25	110 (66.3)
> 25	56 (33.7)
History of using herbal remedies	
Yes	166 (100.0)
Economic status	
Enough for living expenses	133 (80.1)
Insufficient for living expenses	33 (19.9)
Sources of information on herbal remedies^∗^	
Family/friends	24 (14.5)
Gynecologist	15 (9.0)
Other pregnant women	15 (9.0)
Mass media	12 (7.2)

^∗^Respondents could select more than one option.

### 3.2. Prevalence of Herbal Self‐Medication

All participants (*n* = 166, 100%) had a history of using herbal remedies. The prevalence of herbal self‐medication during the current pregnancy was 100% (*n* = 166).

#### 3.2.1. Sources of Information

The most common sources of information regarding herbal remedies were family/friends (*n* = 24, 14.5%), obstetricians/gynecologists (*n* = 15, 9.0%), and other pregnant women (*n* = 15, 9.0%) (Table 1).

#### 3.2.2. Frequency of Herbal Remedies Used

**FIGURE 1:**
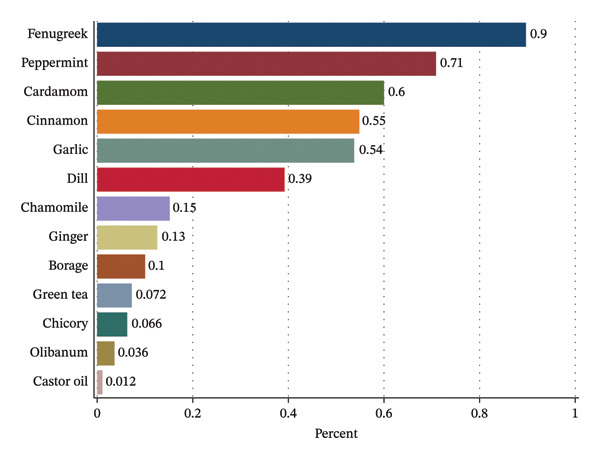
Prevalence of herbal remedies consumed by pregnant women.

The most commonly used herbal remedies during pregnancy were fenugreek (*n* = 149, 89.8%), mint (*n* = 118, 71.1%), and cardamom (*n* = 100, 60.2%) (Figure 1).

#### 3.2.3. Reasons for Use

The most common reasons for using herbal remedies were nausea and vomiting (*n* = 21, 12.7%), prevention of neonatal jaundice (*n* = 15, 9%), and respiratory problems (*n* = 11, 6.6%) (Table 2).

**TABLE 2:** The reasons for using herbal remedies during pregnancy.

Reasons	*n* (%)
Nausea and vomiting	21 (12.7)
Prevention of jaundice in neonates	15 (9.0)
Respiratory problems	11 (6.6)
Skin problems	10 (6.0)
Backache	6 (3.6)
Digestive problems	6 (3.6)
Increasing neonate’s IQ^∗^	3 (1.8)

*Note:* Respondents could select more than one option.

^∗^Intelligence quotient.

#### 3.2.4. Perceived Advantages and Motivations

The findings indicate that traditional reasons (*n* = 63, 38.0%), perceived greater effectiveness compared to pharmaceutical drugs (*n* = 50, 30.1%), and the belief that herbal remedies lack side effects (*n* = 47, 28.3%) were the three primary motivations for the use of herbal remedies among pregnant women. Additionally, easy accessibility (*n* = 20, 12.0%), perceived safety during pregnancy (*n* = 12, 7.2%), affordability (*n* = 9, 5.4%), recommendations from others (*n* = 29, 17.5%), and cultural reasons (*n* = 10, 6%) were also cited as contributing factors.

#### 3.2.5. Frequency and Types of Side Effects

**FIGURE 2:**
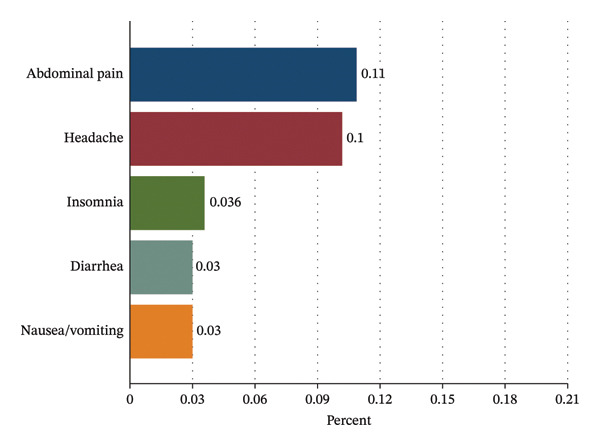
Side effects of using herbal compounds during pregnancy.

Approximately 30.7% of the pregnant women (*n* = 51) reported experiencing side effects after using herbal remedies (Table 1). The most common side effects were abdominal pain (*n* = 18, 10.8%), headache (*n* = 17, 10.2%), and insomnia (*n* = 6, 3.6%) (Figure 2).

#### 3.2.6. Perceived Effectiveness

Most participants (*n* = 96, 57.8%) rated the effectiveness of herbal remedies as moderate, and 27.1% (*n* = 45) rated it as high.

#### 3.2.7. Satisfaction Level

Most participants (*n* = 51, 30.7%) reported moderate satisfaction, and approximately 28.9% (*n* = 48) reported low satisfaction with herbal self‐medication.

## 4. Discussion

Self‐medication with herbal medicines during pregnancy, despite its potential risks, remains common across many societies [19, 20]. The present study was conducted to investigate the prevalence of this behavior among pregnant women in Kermanshah. The findings revealed that all participating pregnant women (100%) had used herbal medicines without a physician’s prescription during pregnancy. Recent evidence from different countries indicates that self‐medication during pregnancy continues to represent a major global public health challenge. For instance, Alhumaid et al. reported a prevalence of 36.5% in Saudi Arabia [21], whereas Akande‐Sholabi et al. reported a prevalence of 69.0% in Nigeria [22]. Similarly, Obiajunwa et al. demonstrated that more than half of pregnant women in southeastern Nigeria practiced self‐medication during pregnancy [23]. Consistent with these findings, Jovanović and Vulić reported that 69.5% of pregnant women in Croatia engaged in self‐medication [24]. Although this behavior was predominantly related to dietary supplements and over‐the‐counter medications, herbal medicine use was also reported as part of this pattern (6.29%), highlighting the persistence of self‐medication behaviors during pregnancy even in countries with more advanced healthcare systems [24]. Previous studies have reported varying prevalence rates of herbal self‐medication during pregnancy, including 32.2% in Tehran, Iran [1], 49.2% in southwestern Ethiopia [11], 22.6% in northern Ethiopia [20], and 20% in São Paulo, Brazil [25]. Cultural diversity, social and religious contexts, accessibility of herbal medicines, financial status, differences in sample size, and variations in study design may all contribute to these discrepancies in prevalence rates [5, 11, 20, 25]. Feyisa et al. further demonstrated that younger maternal age, rural residence, lower educational level, first pregnancy, and a previous history of self‐medication increased the likelihood of engaging in this behavior [10]. These findings underscore the important role of demographic and socioeconomic factors in shaping self‐medication practices. Given the cultural and religious context of Kermanshah, the widespread perception that herbal medicines are “natural” and therefore harmless may encourage pregnant women to use these products without consulting healthcare professionals [8, 26]. Nevertheless, the high prevalence of herbal medicine use during pregnancy, coupled with insufficient evidence regarding their safety and adverse effects, may pose substantial risks to both maternal and fetal health [26]. Therefore, healthcare providers need to educate pregnant women from the early stages of pregnancy, based on scientific evidence, about the potential risks associated with self‐medication using herbal medicines [27].

The findings of the present study also demonstrated that most pregnant women obtained information regarding herbal medicine use from their social networks, particularly family members and friends. This finding is consistent with previous studies. For example, a study conducted in southwestern Ethiopia showed that 49.3% of pregnant women received information about herbal medicine use from family and friends [11]. Similarly, a Brazilian study reported that 61.9% of pregnant women who practiced self‐medication did so based on recommendations from relatives and friends [25]. Comparable findings were reported by Alhumaid et al. who found that previous physician prescriptions and prior personal experiences were major contributors to self‐medication among Saudi pregnant women [21]. Shaibani et al. demonstrated that obstetricians and gynecologists were the primary source of medication‐related information among pregnant women in Libya [21]; however, awareness regarding contraindicated medications during pregnancy remained inadequate. Likewise, Jovanović and Vulić reported that although physicians and formal healthcare sources played an important role in improving pregnant women’s awareness, a considerable proportion of women still relied on public information and informal sources for medication‐related decision‐making [24]. Consequently, the authors emphasized the necessity of strengthening educational programs and improving access to reliable medication‐related information during pregnancy. Obiajunwa et al. further demonstrated that cultural beliefs and previous pregnancy experiences significantly influenced medication‐related decisions among pregnant women [23]. Collectively, these findings indicate that personal experiences and informal social recommendations play a central role in shaping self‐medication behaviors. Identifying pregnant women’s information sources regarding the type and dosage of herbal medicines used is critically important, since recommendations from relatives and friends may expose both mothers and fetuses to serious health risks [28]. These findings highlight the importance of health education during pregnancy delivered by healthcare professionals [27]. Alhazmi et al. additionally reported that only 37.5% of women had adequate awareness regarding the risks of self‐medication [29]. Nurses and midwives, as frontline healthcare providers, play a pivotal role in delivering accurate information to pregnant women and their caregivers [30]. Increasing women’s awareness regarding the risks of herbal self‐medication and the importance of consulting healthcare professionals—including physicians, nurses, and midwives—may substantially reduce this high‐risk behavior. Educational workshops, structured counseling programs, and the use of social media platforms to disseminate evidence‐based educational content may represent effective strategies in this regard [30, 31].

The present study further revealed that most pregnant women resorted to herbal self‐medication due to traditional beliefs and the perception that herbal medicines are safer and more effective than conventional pharmaceuticals. This finding is consistent with studies conducted worldwide. Jovanović and Vulić similarly demonstrated that a large proportion of pregnant women believed that even over‐the‐counter medications and dietary supplements were harmless during pregnancy, despite inadequate awareness regarding the potential consequences of unsupervised medication use. Notably, 94.5% of participants emphasized the need for improved education regarding safe medication use during pregnancy [24]. Noelia et al. reported that women using herbal medicines in Catalonia were more likely to perceive herbal remedies as safer than chemical medications [32]. Likewise, Akande‐Sholabi et al. found that many women considered their illnesses mild and manageable without medical consultation, emphasizing the role of personal perceptions in self‐medication behavior [22]. For instance, approximately half of pregnant women in southern Ethiopia identified fewer side effects and greater effectiveness as the primary reasons for herbal self‐medication [11]. In Brazil, 20.6% of pregnant women reported that culture and tradition were the main motivations for using herbal medicines [25]. Similarly, in Palestine, 67.5% of pregnant women identified the perceived safety of herbal medicines compared with pharmaceutical drugs as the primary reason for their use [2]. Nevertheless, it should be acknowledged that the relative importance of factors influencing herbal self‐medication among pregnant women may vary across different geographical and cultural settings. For example, in Gondar, 54.8% of pregnant women cited affordability and easy accessibility as the principal reasons for herbal medicine use. In contrast, only 12.4% attributed such use to family traditions and cultural beliefs [33]. Iranian studies have likewise demonstrated that pregnant women often use herbal medicines based on recommendations from family members and friends rather than solely due to beliefs regarding their superiority over conventional medications [34]. Therefore, in addition to beliefs regarding greater efficacy and safety, factors such as social recommendations, accessibility, and low cost may also substantially contribute to herbal self‐medication during pregnancy.

The present study identified fenugreek, mint, cardamom, and cinnamon as the most commonly used herbal medicines among pregnant women. This finding is partially consistent with reports from other geographical regions, although some variations were observed. For example, in southwestern Ethiopia, *damakasse*, ginger, and garlic were reported as the most frequently used herbs among pregnant women [11]. Similarly, in Oman, saffron, fenugreek, verbena, brown sugar, and castor oil were among the most commonly used substances during pregnancy. However, in 76% of cases, these products were primarily used to induce labor and enhance uterine contractions [35]. Romero et al. (2025) reported widespread use of ginger, chamomile, thyme, and other herbal products in Catalonia [32], whereas Almutairi et al. identified ginger as the most commonly used herbal medicine among pregnant women in Saudi Arabia [36]. These similarities suggest that herbal medicine use during pregnancy is a global phenomenon. Differences in the pattern of herbal medicine use across countries may be attributed to geographical diversity, cultural differences, indigenous traditions, and the availability of specific plant species [20, 37]. Despite the diversity in the types of herbs consumed, the major concern surrounding herbal self‐medication during pregnancy remains their potential adverse effects on maternal and fetal health. Ahmed et al., in a letter to the editor, emphasized that many existing studies fail to comprehensively evaluate dosage, frequency of use, duration of consumption, and the safety classification of herbal medicines, which may ultimately lead to an underestimation of their actual risks [9]. For instance, the use of fenugreek and cinnamon has been associated with an increased risk of preterm labor and miscarriage [2]. Although mint has traditionally been used to alleviate nausea, vomiting, and other gastrointestinal complaints during pregnancy [38, 39], excessive consumption may increase the risk of uterine bleeding, particularly during the first trimester [40], potentially leading to anemia and miscarriage [38]. Furthermore, although cardamom possesses antiemetic properties [41], its unsupervised use during pregnancy—especially during the first and third trimesters, when fetal growth and development are at their most vulnerable stages—may be associated with serious complications, including congenital abnormalities [1, 27]. These findings collectively underscore the critical need for evidence‐based counseling regarding the safe use of herbal medicines during pregnancy. Given the potential risks associated with herbal self‐medication during pregnancy, educating pregnant women and their families about the adverse effects of these products and the importance of consulting healthcare professionals before consuming any herbal medicine is of paramount importance.

The findings of this study also demonstrated that nausea and vomiting were the most common symptoms prompting pregnant women to use herbal medicines. This observation is consistent with previous studies. For example, a study conducted in southwestern Ethiopia found that 30% of pregnant women who practiced self‐medication used herbal medicines primarily to alleviate nausea and vomiting [11]. Similarly, a Brazilian study reported that 58.7% of pregnant women who consumed herbal products did so to manage nausea and vomiting during pregnancy [25]. Almutairi et al. and Romero et al. also identified ginger as one of the most frequently used herbal remedies for pregnancy‐related nausea, suggesting a global pattern in the use of herbal medicines for gastrointestinal symptoms during pregnancy [32, 36]. The tendency to use herbal remedies for nausea and vomiting may be explained by the high prevalence of these symptoms during pregnancy as a consequence of maternal physiological changes [11, 20]. Nevertheless, despite the traditional use of certain herbal medicines for gastrointestinal discomforts such as nausea and vomiting [1], pregnant women should consult healthcare professionals before consuming any herbal product [20]. This issue is particularly important because many herbal products are perceived as inherently safe despite limited scientific evidence regarding their fetal safety profiles. Given the potential risks of herbal medicines for both maternal and fetal health, emphasizing the importance of consultation with healthcare providers before use during pregnancy is essential.

In the present study, abdominal pain and headache were among the adverse effects reported by pregnant women who practiced herbal self‐medication. This finding is in line with previous studies addressing the potential side effects of herbal medicine use during pregnancy. For example, a study conducted in Ghana found that 52% of pregnant women using herbal medicines experienced abdominal pain and diarrhea, while 20% reported dizziness [19]. Likewise, in northern Ethiopia, most pregnant women believed that herbal medicines could cause adverse outcomes and even result in maternal or neonatal death [20]. However, findings regarding the adverse effects of herbal medicines during pregnancy have not been entirely consistent across studies. For instance, a study conducted in Palestine reported that 95.9% of pregnant women who used herbal medicines experienced no side effects [2]. Similarly, in the Ibadan prospective cohort study in Nigeria, although no significant association was observed between herbal medicine use and adverse pregnancy outcomes, higher rates of macrosomia, birth asphyxia, and postpartum hemorrhage were reported among women who used unregulated medications and herbal products [26]. These inconsistencies highlight the urgent need for comprehensive investigations evaluating the safety and adverse effects of herbal medicines during pregnancy. Unfortunately, robust scientific evidence regarding the safety and efficacy of many herbal medicines remains limited; therefore, there is a pressing need for well‐designed clinical studies to address this critical knowledge gap [25, 27].

In the current study, fewer than one‐third of pregnant women considered herbal medicine use during pregnancy to be effective, and only approximately 30% reported high satisfaction with this therapeutic approach. This level of satisfaction was considerably lower than that reported in several previous studies. For example, in Ghana, 56% of pregnant women considered herbal medicines effective [19]. Similarly, studies conducted in northern and northwestern Ethiopia reported satisfaction rates of approximately 62% and 57%, respectively, regarding the effectiveness of herbal medicines during pregnancy [12, 20]. In Gondar, Ethiopia, the satisfaction rate among pregnant women using herbal medicines was reported to be 31.1% [33]. Variations in satisfaction levels across studies may be attributable to several factors. One possible explanation is the difference in the types of herbal products used and their methods of administration. In addition, factors such as previous maternal experiences regarding the effectiveness of herbal medicines in treating illnesses, educational level, and awareness regarding herbal medicines and their adverse effects, as well as access to healthcare services and alternative therapeutic options, may influence women’s perceptions and evaluations of herbal medicine effectiveness and consequently affect satisfaction levels [19, 20, 42–44]. Studies such as Akande‐Sholabi et al. have shown that a discrepancy often exists between women’s awareness and their actual behaviors, with many pregnant women continuing to engage in high‐risk practices despite having some degree of knowledge regarding potential harms [22]. Alhazmi et al. also emphasized that lower educational attainment and poor socioeconomic status were associated with increased self‐medication behaviors [29]. These findings emphasize that awareness alone may be insufficient to change behavior unless accompanied by accessible counseling services and culturally tailored educational interventions. In this context, Jovanović and Vulić similarly reported that a considerable proportion of pregnant women acknowledged the importance of education regarding safe medication use during pregnancy and expressed the need for improved educational programs and better access to medication‐related information, further reflecting the gap between awareness and actual medication‐use behaviors during pregnancy [24].

### 4.1. Strengths and Limitations

Adhering to the STROBE guidelines is a significant strength of this study. This adherence enhances the accuracy and transparency of reporting methods and findings, ultimately bolstering the study’s credibility. The sample size is deemed sufficient for this study. The absence of attrition also highlights the researchers’ meticulousness and rigorous follow‐up during data collection, further strengthening the study’s quality. Finally, using a validated questionnaire enhances the accuracy and reliability of the collected data.

This study, while aiming to provide a comprehensive overview of herbal remedies and self‐medication among pregnant women, has limitations that should be considered when interpreting the findings. Self‐medication is a multifaceted phenomenon influenced by various factors, including geographical location, culture, education level, socioeconomic status, and access to healthcare services. Therefore, generalizing these findings to other populations should be done cautiously. This study did not examine details regarding the consumed herbal remedies, such as dosage, preparation methods, and duration of use. This information is crucial for a more accurate assessment of the potential risks and benefits of herbal self‐medication during pregnancy. Data on herbal remedy use were primarily collected through self‐reported questionnaires, which may have influenced the results due to recall bias, social desirability bias, or a lack of precise knowledge among mothers regarding the composition of the consumed plants.

## 5. Conclusion

Self‐medication with herbal remedies is a common practice among pregnant women globally. The findings of this study indicate that herbal self‐medication, particularly with fenugreek, mint, cardamom, and cinnamon, is prevalent among pregnant women residing in Kermanshah. Considering the serious risks associated with the unsupervised use of these substances, including miscarriage, macrosomia, birth asphyxia, and postpartum hemorrhage, the need for education and awareness‐raising among pregnant women regarding the dangers of this practice is paramount. Further research into the safety and efficacy of herbal remedies during pregnancy, as well as the factors influencing decision‐making among pregnant women and their caregivers, can help improve the quality of prenatal care and reduce complications from the unsupervised use of herbal remedies.

## Author Contributions

Mandana Sayadi Mank‐Halati, Shahab Rezaeian, Maryam Janatolmakan, Sahar Omidi, and Alireza Khatony contributed to designing the study. Mandana Sayadi Mank‐Halati and Sahar Omidi collected the data, and the data were analyzed by Shahab Rezaeian. The final report and manuscript were written by Mandana Sayadi Mank‐Halati, Shahab Rezaeian, Maryam Janatolmakan, Sahar Omidi, and Alireza Khatony.

## Funding

The research was financially supported by Kermanshah University of Medical Sciences under Grant No. 50003314, with Alireza Khatony as the grant recipient.

## Disclosure

All the authors read and approved the version for submission.

## Ethics Statement

The study was approved by the ethics committee of Kermanshah University of Medical Sciences with the Code IR.KUMS.REC.1402.316. Written informed consent was obtained from all participants. All experimental protocols involving human subjects adhered to the relevant national/international/institutional guidelines or the Declaration of Helsinki.

## Consent

The authors have nothing to report.

## Conflicts of Interest

The authors declare no conflicts of interest.

## Data Availability

The data that support the findings of this study are available on request from the corresponding author. The data are not publicly available due to privacy or ethical restrictions.
